# Efficacy, safety and predictors of combined fruquintinib with programmed death-1 inhibitors for advanced microsatellite-stable colorectal cancer: A retrospective study

**DOI:** 10.3389/fonc.2022.929342

**Published:** 2022-08-31

**Authors:** Weijie Zhang, Zhongyue Zhang, Shitong Lou, Donghui Li, Zhijun Ma, Lei Xue

**Affiliations:** Department of Oncology, The First Affiliated Hospital of Zhengzhou University, Zhengzhou, China

**Keywords:** fruquintinib, PD-1 inhibitors, microsatellite-stable (MSS), colorectal cancer (CRC), predictors, retrospective study

## Abstract

**Background:**

Research findings have revealed that combining anti-angiogenesis inhibitors with programmed death-1(PD-1) inhibitors can reverse the immunosuppressive tumor microenvironment and enhance the antitumor immune response. To explore the therapeutic options for breaking immune tolerance in microsatellite stability (MSS) or mismatch repair-proficiency (pMMR) advanced colorectal cancer (CRC), we assessed the efficacy, safety and predictors of the fruquintinib and PD-1 inhibitors combination in patients with MSS/pMMR advanced CRC in a real-world environment.

**Methods:**

We conducted a single-center retrospective study by collecting relevant data on patients with MSS/pMMR advanced CRC who received fruquintinib coupled with PD-1 inhibitors in the First Affiliated Hospital of Zhengzhou University between August 2019 and November 2021, focusing on progression-free survival.

**Results:**

We enrolled 110 eligible patients in this study between August 2019 and November 2021. At the deadline (January 20, 2022), 13 patients had objective responses. The objective response rate was 11.8% (13/110, 95% confidence interval [CI]: 6.4-18.2), the disease control rate was 70.0% (82/110, 95% CI: 60.9-78.2), and the progression-free survival was 5.4 months (95% CI: 4.0-6.8). Liver metastases (hazard ratio [HR]: 0.594, 95% CI: 0.363-0.973, P<0.05), alkaline phosphatase elevation (ALP>160U/L) (HR: 0.478, 95%CI: 0.241-0.948, P<0.05), fibrinogen elevation (FIB>4g/L) (HR: 0.517, 95% CI: 0.313-0.855, P<0.05), and an increase in the ALP level from the baseline after treatment (HR: 1.673, 95% CI: 1.040-2.690, P<0.05) were negative predictors of the progression-free survival. A total of 101 of 110 patients experienced treatment-related adverse events, including 14 who experienced grade 3 or above treatment-related adverse events, and no treatment-related deaths occurred. Hypertension was the most frequently encountered grade 3 treatment-related adverse event.

**Conclusion:**

Fruquintinib combined with PD-1 inhibitors has antitumor activity and manageable safety in treating patients with MSS/pMMR advanced CRC. Liver metastases, ALP level and FIB level might be a prediction of the patient response to this therapy.

## Introduction

Colorectal cancer (CRC) is the third most prevalent cancer and the second largest cause of mortality globally (1). Due to the lack of specificity in its early diagnosis, the majority of patients with the condition are diagnosed at an advanced stage. Chemotherapy remains the standard treatment for CRC despite its disadvantages such as apparent systemic adverse effects, low selectivity, and drug concentration at the tumor site. Immunotherapy has shown promising outcomes in treating various malignant tumors in recent years. The approved immune checkpoint inhibitors (ICIs) for CRC, pembrolizumab and nivolumab ± ipilimumab, are only suited for patients with microsatellite instability-high (MSI-H) or mismatch repair-deficient (dMMR) (2, 3), whereas 95% of CRC patients have microsatellite stability (MSS) or mismatch repair-proficiency (pMMR), and single-agent ICIs are ineffective. The objective response rate (ORR) of pembrolizumab in the MSS/pMMR CRC cohort in the KEYNOTE-016 study was 0% (4), and advanced patients have fewer options for later-line treatment.

Fruquintinib is a small-molecule tyrosine kinase inhibitor that targets the vascular endothelial growth factor receptor (VEGFR) 1, 2, and 3, and it is highly selective and potent. The FRESCO study found that fruquintinib extended the median overall survival (OS) (9.3 months vs. 6.6 months) and progression-free survival (PFS) (3.7 months vs. 1.8 months) when compared to placebo (5). The Food and Drug Administration has approved fruquintinib as a third-line treatment for metastatic CRC (mCRC).

According to growing evidence, anti-angiogenic therapy combined with immunotherapy strengthens the vascular normalization and immune reprogramming, reversing the immunosuppressive tumor microenvironment (TME) and inducing a long-lasting antitumor immune response in the body (6, 7). In a case report, after failing multiple lines of treatment, a late-stage patient who received fruquintinib plus sintilimab experienced a quick remission. The antitumor activity and the ability to reprogram the immunosuppressive TME of fruquintinib plus PD-1 inhibitors were confirmed in mouse experiments (8). According to an American Society of Clinical Oncology (ASCO) study published in 2020, the ORR of fruquintinib plus sintilimab in the treatment of mCRC was much higher than that of single-agent fruquintinib (15.4% vs. 4.9%) (9). The Phase Ib/II study of the fruquintinib and sintilimab combination for advanced CRC showed that the ORR was 27.3% and the PFS was 6.9 months in the fruquintinib 5 mg intermittent-treatment group according to the 2021 ASCO Meeting Abstract (10). These findings suggest that fruquintinib coupled with PD-1 inhibitors has a promising application in advanced CRC, providing compelling evidence of the clinical application of combined therapy.

These findings suggest novel approaches to treating MSS/pMMR advanced CRC. However, clinical trial data are limited due to the strict inclusion criteria that are implemented. In the real world, the efficacy and safety of fruquintinib coupled with PD-1 inhibitors in treating MSS/pMMR mCRC are unknown, and few studies have reported efficacy predictors for this treatment. Therefore, we designed this retrospective study.

## Patients and methods

### Patients

**Figure 1:**
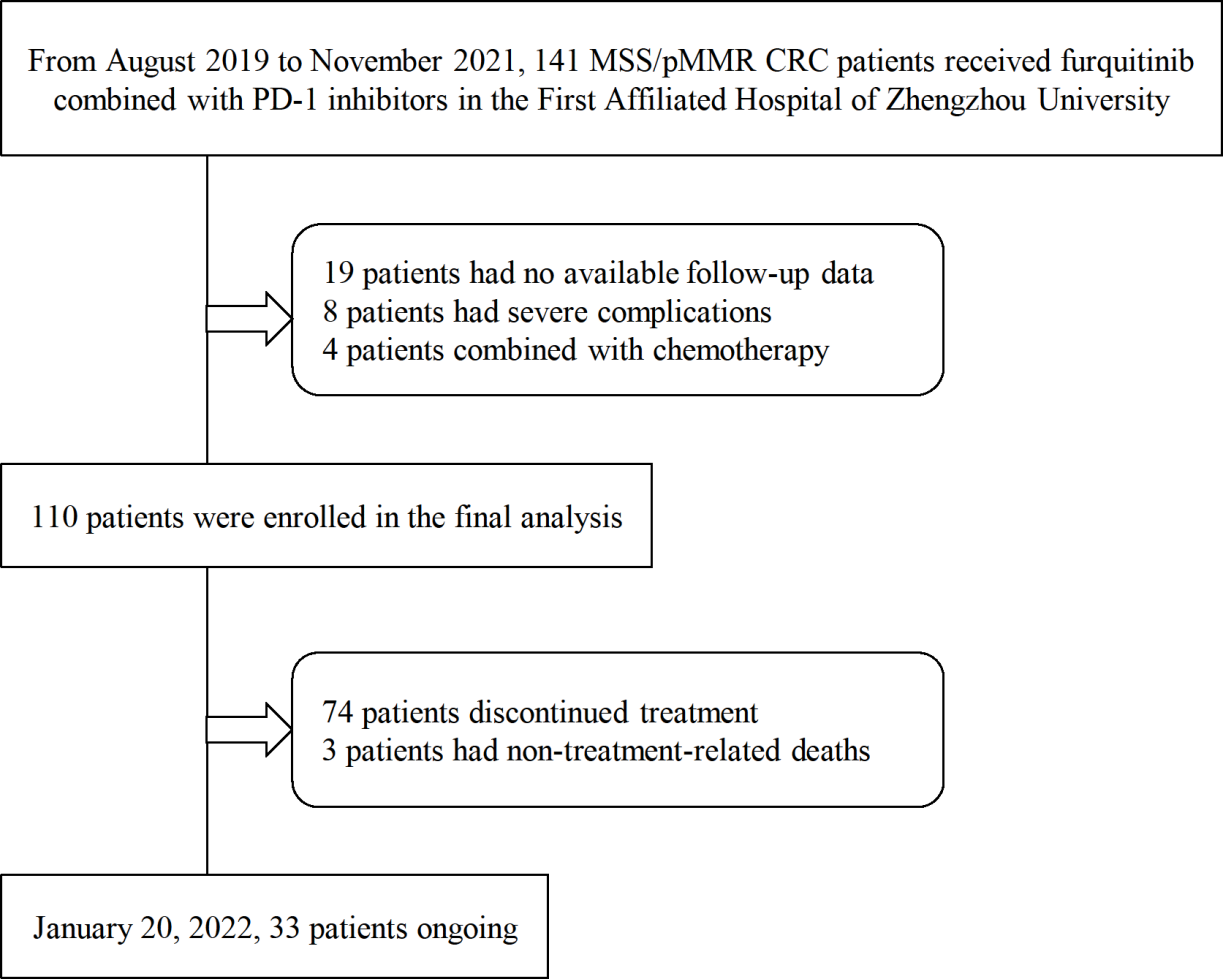
The patient selection process for the retrospective cohort. MSS/pMMR, microsatellite stability or mismatch repair-proficiency; CRC, colorectal cancer; PD-1, programmed death-1.

From August 2019 to November 2021, patients with MSS/pMMR advanced CRC receiving fruquintinib plus PD-1 inhibitors at the First Affiliated Hospital of Zhengzhou University were included in this single-center retrospective study. Out of the 141 eligible patients, 31 patients were excluded, 19 had no available follow-up data, 4 received treatment that was combined with other chemotherapy drugs, and 8 had severe underlying diseases or other tumor complications. The data of the remaining 110 patients were analyzed. A flow chart of the patient selection procedure is shown in **Figure 1**. The main inclusion criteria were: 1. advanced or metastatic CRC confirmed by histology or cytology; 2. MSS/pMMR confirmed by tumor genetic testing in a local laboratory; 3. an Eastern Cooperative Oncology Group Performance Status (ECOG PS) score of <3; 4. the patient’s disease progressed after ≥2nd-line therapy; 5. the presence of measurable lesions that meet the Response Evaluation Criteria for Solid Tumors Version 1.1 (RECIST V1.1); 6. sufficient reserves of the bone marrow, liver, kidney organ functions and the coagulation function. The key exclusion criteria were as follows: 1. active or previous chronic or recurring autoimmune illness; 2. severe comorbidity; 3. a definitive diagnosis of hereditary CRC syndrome. The data are all from the medical record system of the First Affiliated Hospital of Zhengzhou University. The patient’s clinical features, gene status, laboratory results, tumor responses, and treatment-related adverse events (TRAEs) were collected and analyzed. **Table 1** summarizes the baseline characteristics of the study participants.

**Table 1:** Baseline Characteristics.

Characteristic	Patients (n=110)
**Age (years)**	
Median age (range)	53.0 (22–81)
<65 years	91 (82.7%)
≥65 years	19 (17.3%)
**Gender**	
Male	63 (57.3%)
Female	47 (42.7%)
**Baseline ECOG PS**	
0	31 (28.2%)
1	67 (60.9%)
2	12 (10.9%)
**Site of primary tumor**	
Left-side	86 (78.2%)
Right-side	24 (21.8%)
**Site of metastases**	
Liver	60 (54.5%)
Lung	64 (58.2%)
Lymph node	79 (71.8%)
Peritoneum	25 (22.7%)
Other	47 (42.7%)
**Number of organs with metastases**	
<2	21 (19.1%)
≥2	89 (80.9%)
**Previous therapies**	
Fluoropyrimidines	109 (99.1%)
Platinum agents	107 (97.3%)
Irinotecan	91 (82.7%)
Antiangiogenic therapy	98 (89.1%)
Immunotherapy	11 (10.0%)
Antiangiogenic plus immunotherapy	9 (8.2%)
Surgery	85 (77.3%)
Radiotherapy	10 (9.1%)
**Prior treatment lines**	
≤3	32 (29.1%)
>3	78 (70.9%)
**Gene mutation status**	
*RAS/BRAF* wild	28 (25.5%)
*RAS* mutant	54 (49.1%)
*BRAF* mutant	6 (5.4%)
Unknown	22 (20.0%)
**ALP**	
> 160U/L	12 (10.9%)
≤ 160U/L	98 (89.1%)
**GGT**	
> 50U/L	43 (39.1%)
≤ 50U/L	67 (60.9%)
**LDH**	
> 245U/L	55 (50.0%)
≤ 245U/L	55 (50.0%)
**D-Dimer**	
> 0.3mg/L	55 (50.0%)
≤ 0.3 mg/L	55 (50.0%)
**FIB**	
> 4g/L	31 (28.2%)
≤ 4g/L	79 (71.8%)
**TMB**	
TMB-H	8 (7.3%)
TMB-L	25 (22.7%)
Unknow	77 (70.0%)

ECOG PS, Eastern Cooperative Oncology Group Performance Status; ALP, alkaline phosphatase; GGT, glutamyl transpeptidase; LDH, lactate dehydrogenase; FIB, fibrinogen; TMB, tumor mutational burden; TMB-H, high tumor mutational burden; TMB-L, low tumor mutational burden.

Site of primary tumor, Left-side: distal 1/3 of transverse colon, descending colon, sigmoid colon and rectum, Right-side: proximal 2/3 transverse colon, cecum, ascending colon.

Site of metastases, others: 23 had pelvic metastases, 12 had bone metastases, 3 brain metastases, 5 kidney metastases, 4 bladder metastases, 5 ureteral metastases, 2 spleen metastases, 1 gallbladder metastases, 3 soft tissue metastases, 1 penis metastases, 1 chest wall metastases.

Biochemical and coagulation indicators used the upper limit of normal as the cutoff value.

TMB-H defined as ≥10 mut/Mb.

### Treatment methods

Patients received fruquintinib at a dose of 3–5 mg once a day for 14/21 days, every 28 days. Patients received PD-1 inhibitors including sintilimab, camrelizumab, toripalimab, tislelizumab, and pembrolizumab. Patients received fixed doses of sintilimab, camrelizumab, tislelizumab, and pembrolizumab (200 mg) every 3 weeks, and toripalimab (240 mg) every 3 weeks on the first day of fruquintinib application. To control TRAEs, the dose of fruquintinib was adjusted in some patients; however, the doses of PD-1 inhibitors were not.

### Assessment

According to RECIST V1.1, tumor response was tested using computed tomography every 6–8 weeks. Tumor response to treatment was assessed as complete response (CR: all target lesions disappeared and no new lesions appeared), partial response (PR: the sum of the longest diameters of all target lesions is less than 30% of the baseline), progression (PD: a 20% increase in the longest diameter of all target lesions compared to the minimum, or the appearance of new lesions), and stable (SD: insufficient reduction to achieve PR but insufficient increase to achieve PD). The ORR is the proportion of patients achieving CR and PR, and the disease control rate (DCR) is the proportion of patients achieving CR, PR, and SD. PFS was defined as the time from treatment initiation to either disease progression or death. The time from the treatment of the study until death from any cause was defined as the OS. TRAEs were assessed by the National Cancer Institute Common Terminology Criteria for Adverse Events Version 5.0 (NCI CTCAE V5.0).

### Statistical analysis

The baseline characteristics of CRC patients were described using percentages and median values. Pearson’s chi-square test was used to analyze the effects of patients’ baseline characteristics on the efficacy of combination therapy for statistical analysis. P<0.05 was considered statistically significant. The Kaplan-Meier survival analysis was used to calculate the PFS, and the log-rank test was used to compare subgroups to each other. Hazard ratios (HRs) and confidence intervals (CIs) were calculated using the Cox proportional hazards regression model for variables with P<0.05 in univariate analysis. Statistical Product Service Solutions Version 21.0 and GraphPad Prism Version 9.0 were used for statistical analysis.

## Results

### Patients characteristics and treatment strategies

A total of 110 patients were enrolled from August 2019 to November 2021. The median age was 53 years (range: 22–81). A total of 91 (82.7%) patients were less than 65 years old, and 38 (34.5%) patients were early-onset (<50 years). At the beginning of treatment, 12 (10.9%) patients had an ECOG PS score of 2. At the initial diagnosis, the tumors of 86 (78.2%) patients were located in the left side (distal 1/3 of transverse colon, descending colon, sigmoid colon and rectum) and those of 24 (21.8%) patients were located in the right side (proximal 2/3 transverse colon, cecum, ascending colon). At the start of treatment, 60 (54.5%) patients had liver metastases, 64 (58.2%) had lung metastases, 47 (42.7%) had other organs metastases (23 had pelvic metastases, 12 had bone metastases, 3 brain metastases, 5 kidney metastases, 4 bladder metastases, 5 ureteral metastases, 2 spleen metastases, 1 gallbladder metastases, 3 soft tissue metastases, 1 penis metastases, 1 chest wall metastases), and 89 (80.9%) had at least two metastases. Treatment methods varied, and all patients received ≥2 lines of prior chemotherapy, 98 (89.1%) received anti-angiogenic therapy (mostly bevacizumab and cetuximab), 11 (10.0%) received PD-1 inhibitors, 9 (8.2%) patients received anti-angiogenic drugs combined with PD-1 inhibitors, 85 (77.3%) underwent surgery (including primary tumor and metastases resection), 10 (9.1%) received radiotherapy. All patients had histologically or cytologically confirmed CRC, and the gene test results were MSS/pMMR. A total of 54 (49.1%) patients had a *RAS* mutation, and 6 (5.4%) patients had a *BRAF* mutation. Thirty-three of the 110 patients were tested for tumor mutational burden (TMB), 8 (7.3%) patients showed high-TMB (TMB-H, TMB-H was defined as≥10mut/Mb), and 25 (22.7%) showed low-TMB (TMB-L). We collected the ALP, glutamyl transpeptidase (GGT), lactate dehydrogenase (LDH), D-Dimer, FIB values of 110 patients at baseline and divided patients into two groups using the upper limits of normal values as cutoff values. The baseline characteristics of study participants are shown in **Table 1**.

In this study, a total of five PD-1 inhibitors were used in 110 patients, with the most common ones being sintilimab (46.4%), camrelizumab (35.5%), and toripalimab (13.6%). In all patients, these molecules were combined with fruquintinib, and most of them (92.7%) received fruquintinib at a dose of 5 mg (**Table 2**).

**Table 2:** Programmed death-1 inhibitors and fruquintinib combination strategies.

Drug	n (%)
**Programmed death-1 inhibitors**	
Sintilimab	51 (46.4%)
Camrelizumab	39 (35.5%)
Toripalimab	15 (13.6%)
Tislelizumab	4 (3.6%)
Pembrolizumab	1 (0.9%)
**Fruquintinib**	
Fruquintinib 3mg	7 (6.4%)
Fruquintinib 4mg	1 (0.9%)
Fruquintinib 5mg	102 (92.7%)

### Clinical efficacy

**Figure 2:**
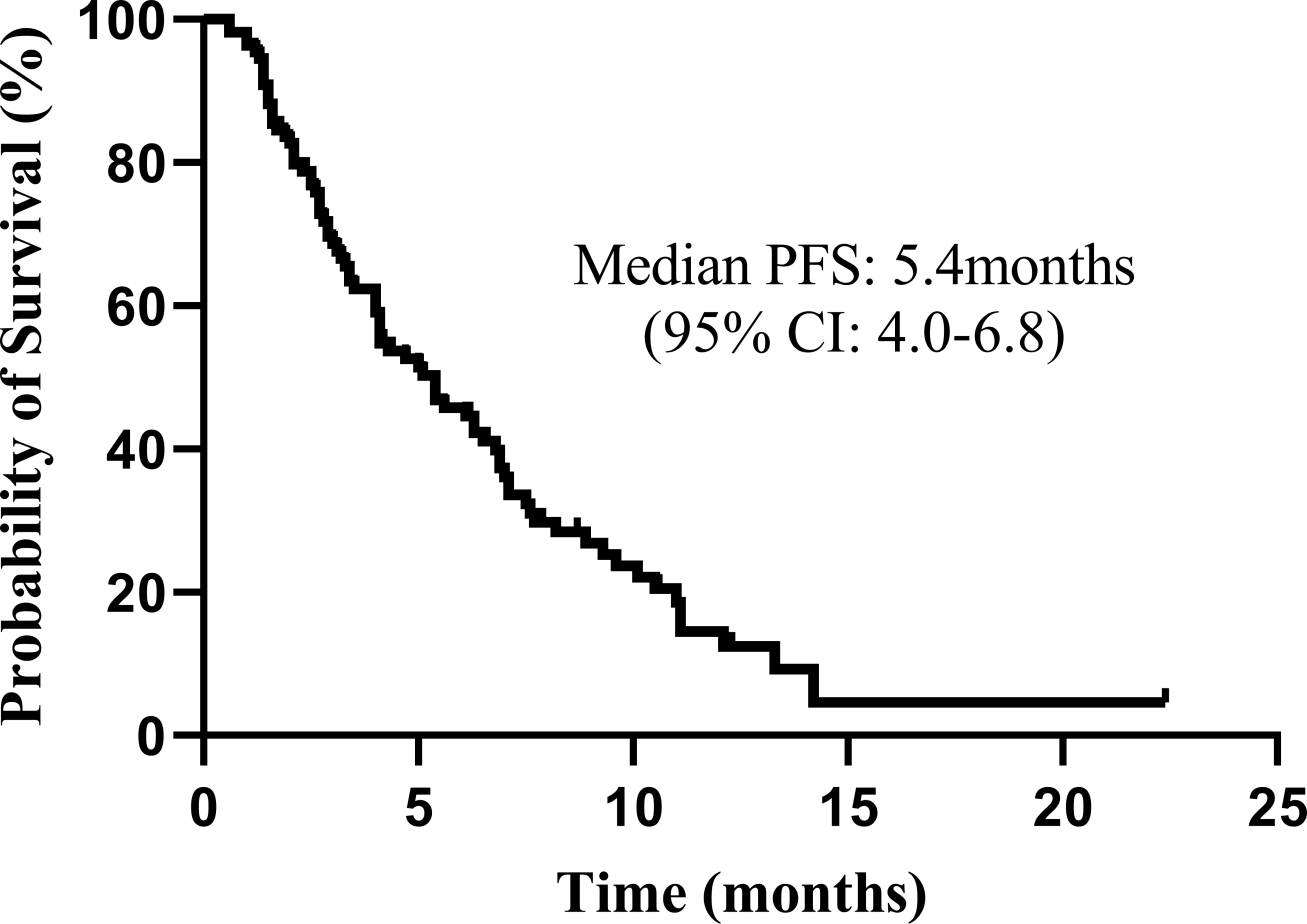
Kaplan–Meier plot for the total population (n = 110). The progression-free survival (PFS) from the beginning of the Fruquintinib and programmed death-1 (PD-1) inhibitors combination therapy. CI, confidence interval.

**Figure 3:**
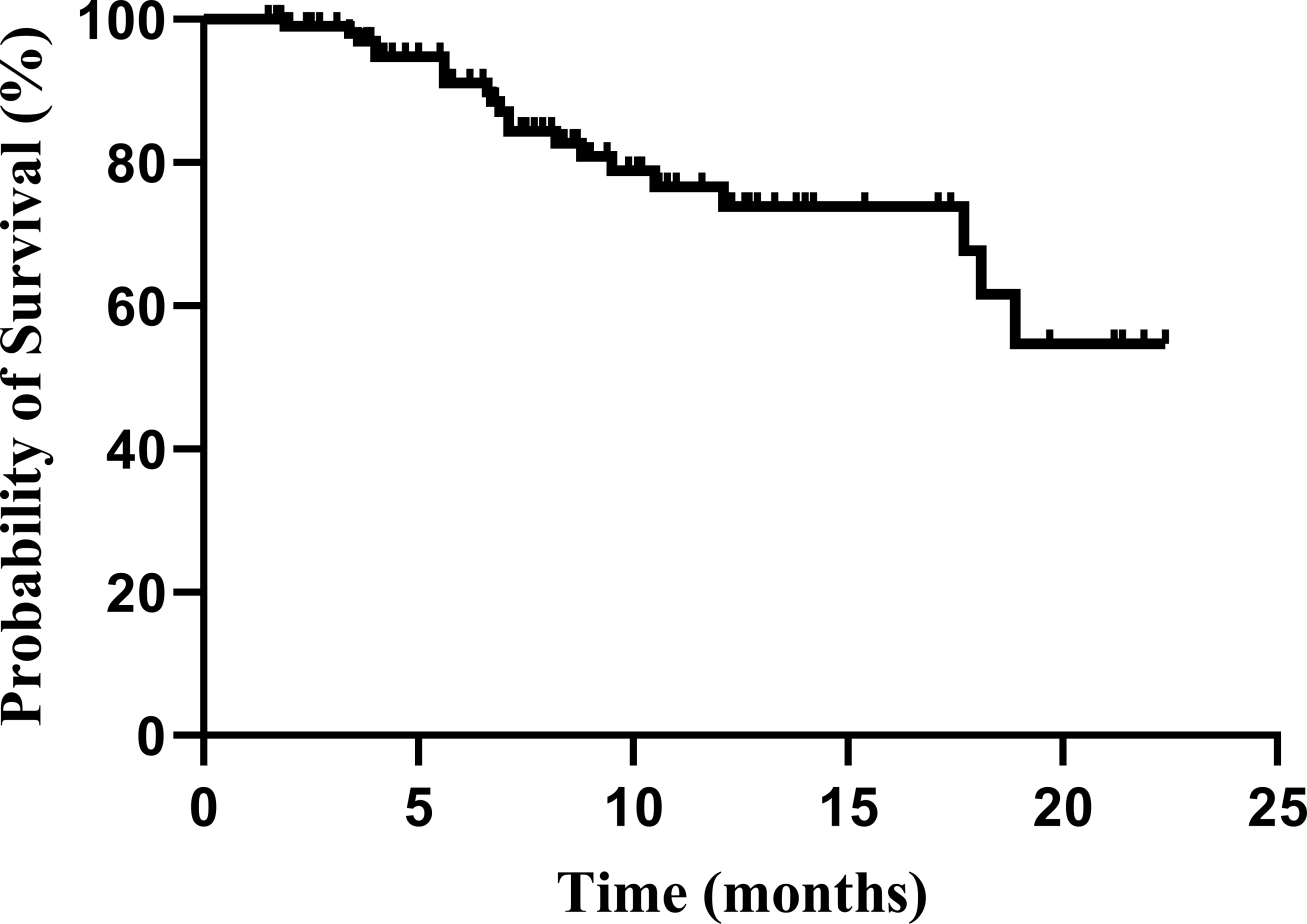
Kaplan–Meier plot for the total population (n = 110). The overall survival (OS) from the beginning of the Fruquintinib and programmed death-1 (PD-1) inhibitors combination therapy.

As of January 20, 2022, 110 patients who received the combination of fruquintinib and PD-1 inhibitors underwent at least one tumor imaging assessment. **Table 3** summarizes the patients’ best responses from the baseline. None of the patients achieved CR, 13 (11.8%) of them achieved PR, and 64 (58.2%) were rated as SD. The ORR was 11.8% (13/110, 95% CI: 6.4-18.2), and the DCR was 70.0% (82/110, 95% CI: 60.9–78.2). The median PFS was 5.4 months (95% CI: 4.0–6.8) (**Figure 2**). The median OS was not determined in our study (**Figure 3**).

**Table 3:** Curative effect evaluation.

	n=110 (%)
Partial response	13 (11.8%)
Stable disease	64 (58.2%)
Progressive disease	33 (30.0%)
Objective response	13 (11.8%) [95%CI:6.4~18.2]
Disease control	82 (70.0%) [95%CI:60.9~78.2]

CI, confidence interval.

No factors, including age, gender, location of the primary tumor, ECOG score, treatment lines, previous treatment methods, the presence or absence of metastases (liver, lung, peritoneum, pelvic, bone), gene mutation status, some biochemical and coagulation indicators (ALP, GGT, LDH, D-Dimer, and FIB) at baseline, were found to have a significant impact on the effectiveness of the fruquintinib and PD-1 inhibitors combination.

**Figure 4:**
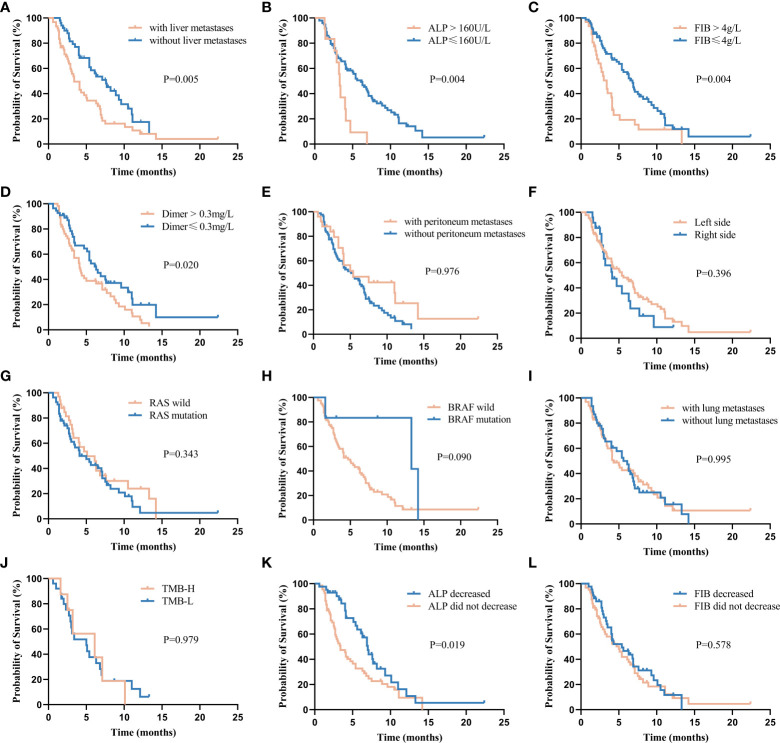
Kaplan–Meier plot for progression-free survival (PFS) stratified by clinical factors, including **(A)** liver metastases, **(B)** alkaline phosphatase (ALP), **(C)** fibrinogen (FIB), **(D)** D-Dimer, **(E)** peritoneum metastases, **(F)** Left-side: distal 1/3 of transverse colon, descending colon, sigmoid colon and rectum, Right-side: proximal 2/3 transverse colon, cecum, ascending colon, **(G)** RAS wild/mutant, **(H)** BRAF wild/mutant, **(I)** lung metastases, **(J)** high tumor mutational burden (TMB-H, ≥10 mut/Mb) / low tumor mutational burden (TMB-L, < 10 mut/Mb), **(K)** changes from baseline in ALP levels after treatment, **(L)** changes from baseline in FIB levels after treatment.

**Figure 5:**
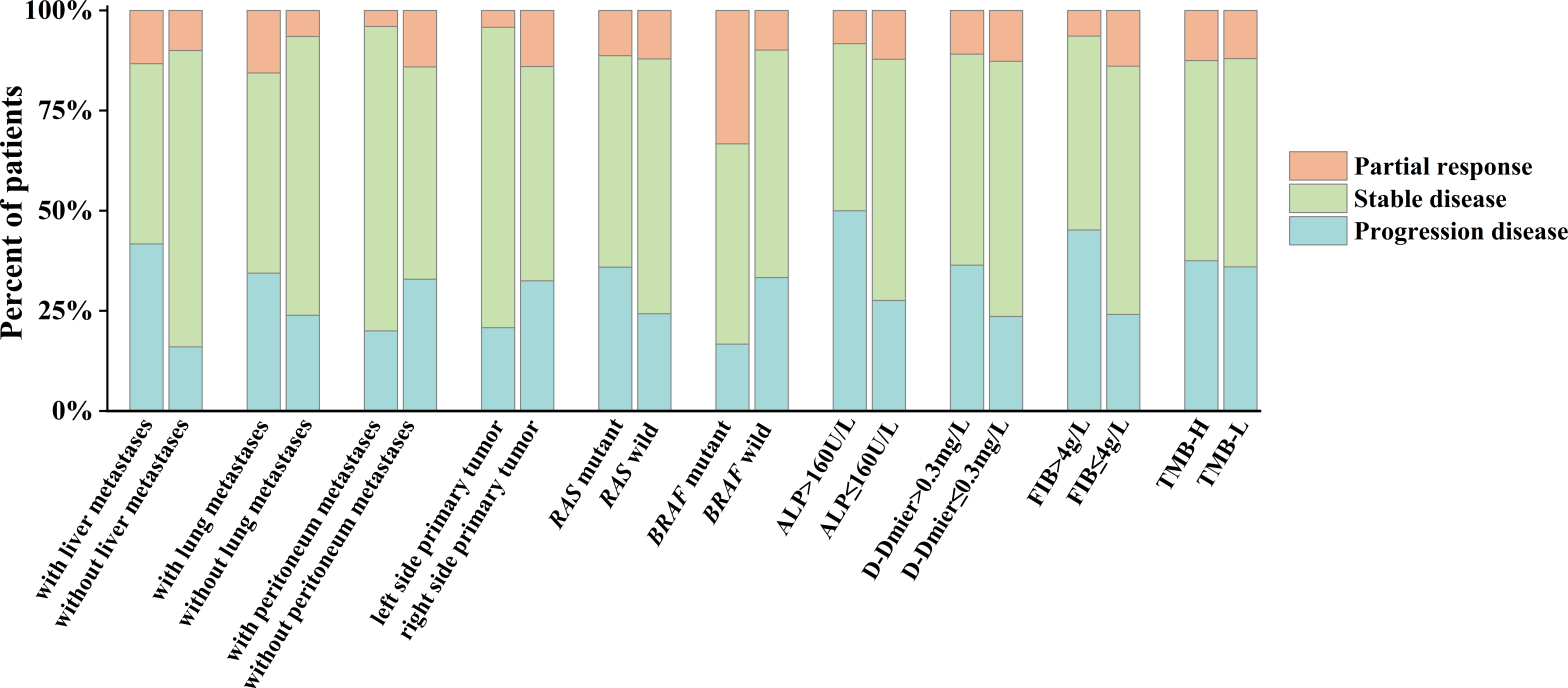
Efficacy in selected subgroups. ALP, alkaline phosphatase; FIB, fibrinogen; TMB-H, high tumor mutational burden (≥10 mut/Mb), TMB-L, low tumor mutational burden (<10 mut/Mb).

The Kaplan-Meier survival analysis found that the PFS of patients with liver metastases was significantly shorter than that of patients without them (PFS: 3.4 [95% CI: 2.3–4.5] months vs. 7.6 [95% CI: 5.3–9.9] months, P<0.05, **Figure 4A** **)**. The patients with ALP>160 U/L had a substantially shorter PFS than those without them (PFS: 3.3 [95% CI: 3.0-3.7] months vs. 6.1 [95% CI: 4.6-7.6] months, P<0.05, **Figure 4B** **)**. The PFS of patients with FIB>4g/L was significantly shorter than that of patients without them (3.4 [95%CI:2.5-4.3] months vs. 6.8 [95%CI:5.8-7.8] months, P<0.05, **Figure 4C** **)**. Patients with D-dimer ≤0.3 mg had longer PFS (PFS: 6.3[95% CI: 4.8–7.8] months vs. 4.1[95% CI: 3.9–4.2] months, P<0.05, **Figure 4D**). The PFS was extended in patients with peritoneum metastases (P=0.976, **Figure 4E**), primary lesions located in the left side (P=0.396, **Figure 4F**), RAS wild (P=0.343, **Figure 4G**), BRAF-mutated (P=0.090, **Figure 4H**) and patients without lung metastases (P=0.995, **Figure 4I**) compared with their corresponding subgroups. In comparison to the TMB-L group, the PFS in the TMB-H group was longer (5.0 [95%CI: 2.0-8.0] months vs. 6.1 [95%CI: 0.0-12.2] months, P=0.979, **Figure 4J**). The comparison of the efficacy of each subgroup is shown in **Figure 5**.

Patients were assessed for ALP and FIB after two cycles of combination therapy and compared with pre-treatment values. The ALP level was decreased in 42 (38.9%) patients and increased in 66 (61.1%) patients. The PFS was 7.0 (95% CI: 5.9-8.0) months and 3.4 (95% CI: 2.2-4.8) months in the decreased and increased ALP level groups (P<0.05, **Figure 4K**). The FIB level was decreased in 43 (40.6%) patients and increased in 63 (59.4%) patients. The PFS was 5.4 (95% CI: 2.8–8.0) months and 5.0 (95% CI: 3.6–6.5) months in the FIB-lowering and FIB-raising groups (P=0.578, **Figure 4L**).

The multivariate Cox proportional hazards regression analysis results showed that liver metastases, ALP level, FIB level, and changes in ALP level after treatment were associated with PFS in fruquintinib coupled with PD-1 inhibitors in MSS/pMMR CRC (P<0.05, **Table 4**).

**Table 4:** Influence of clinical factors on progression-free survival.

	Univariate analysis		Multivariate analysis	
	HR (95% CI)	P value	HR (95% CI)	P value
Age (<65/≥65years)	1.662 (0.904-3.054)	0.096		
Gender (Male/Female)	1.131 (0.722-1.772)	0.587		
Baseline ECOG PS (0/1/2)	1.324 (0.903-1.941)	0.342		
Site of primary tumor (Left side/Right side)	1.262 (0.733-2.171)	0.396		
**Liver metastases (yes/no)**	**0.531 (0.337-0.838)**	**0.005**	**0.594 (0.363-0.973)**	**0.038**
Lung metastases (yes/no)	0.999 (0.639-1.561)	0.995		
Peritoneum metastases (yes/no)	0.992 (0.589-1.672)	0.976		
Pelvic metastases (yes/no)	1.601 (0.908-2.826)	0.098		
Bone metastases (yes/no)	0.817 (0.403-1.653)	0.569		
Number of organs with metastases (≥2/<2)	1.012 (0.582-1.761)	0.966		
Treatment lines (3/>3)	2.280 (0.706-7.366)	0.154		
*RAS* status (wild/mutant)	1.281 (0.764 -2.148)	0.343		
*BRAF* status (wild/mutant)	0.372 (0.113-1.222)	0.090		
Prior antiangiogenic therapy (yes/no)	0.422 (0.170-1.046)	0.052		
Prior immunotherapy (yes/no)	0.945 (0.452-1.978)	0.880		
Prior antiangiogenic plus immunotherapy (yes/no)	0.724 (0.346-1.511)	0.382		
**ALP (>160U/L/≤160U/L)**	**0.390 (0.200-0.757)**	**0.004**	**0.478 (0.241-0.948)**	**0.035**
GGT (>50U/L/≤50U/L)	0.788 (0.329-1.888)	0.475		
LDH (>245U/L/≤245U/L)	1.534 (0.643-3.675)	0.213		
**D-Dimer (>0.3mg/L/≤0.3mg/L)**	**0.584 (0.371-0.918)**	**0.020**	0.783 (0.491-1.247)	0.302
**FIB (>4g/L/≤4g/L)**	**0.503 (0.314-0.807)**	**0.004**	**0.517 (0.313-0.855)**	**0.010**
TMB (TMB-H/TMB-L)	0.988 (0.391-2.493)	0.979		
**Changes in ALP levels (decreased/did not decreased)**	**1.743 (1.091-2.785)**	**0.019**	**1.673 (1.040-2.690)**	**0.034**
Changes FIB levels (decreased/did not decreased)	1.138 (0.723-1.790)	0.578		

ECOG PS, Eastern Cooperative Oncology Group Performance Status; ALP, alkaline phosphatase; GGT, glutamyl transpeptidase; LDH, lactate dehydrogenase; FIB, fibrinogen; TMB, tumor mutational burden; TMB-H, high tumor mutational burden (≥10 mut/Mb); TMB-L, low tumor mutational burden (<10 mut/Mb).

Site of primary tumor, Left-side: distal 1/3 of transverse colon, descending colon, sigmoid colon and rectum, Right-side: proximal 2/3 transverse colon, cecum, ascending colon.

The bold values represent P<0.05, and the difference is statistically significant.

Considering that the difference in PFS caused by ALP and FIB levels may be caused by the metastatic site, we compared the clinical characteristics of ALP>160U/L group and ALP ≤ 160U/L group, FIB>0.4g/L group and FIB<0.4 g/L group, and found that patients with liver metastases had a higher incidence of the elevated ALP level than those without liver metastases (16.7% vs. 4.0%, P=0.034), while none of the characteristics assessed between FIB subgroups were significantly different. In patients without liver metastases, the PFS of ALP>160U/L group and ALP ≤ 160U/L group were 7.7 months and 3.2 months (P=0.052).

There was no significant difference in the efficacy of different PD-1 inhibitors and fruquintinib combinations (sintilimab: PFS: 4.1 [95% CI: 2.5-5.6] months, ORR: 7.8%, DCR: 68.6%; camrelizumab: PFS: 5.6 [95% CI: 3.3-7.9] months, ORR: 7.7%, DCR: 69.2%; tislelizumab: PFS: 6.8 [95% CI: 3.8-9.8] months, ORR: 33.3%, DCR: 73.3%), and no significant difference between different doses of the fruquintinib and PD-1 inhibitors combination (fruquintinib 3mg: PFS: 4.1 [95% CI: 2.5-5.7] months, ORR: 0%, DCR: 85.7%; fruquintinib 5mg: PFS: 5.4 [95% CI: 3.7-7.1] months, ORR: 12.7%, DCR: 68.6%).

### Safety

As of January 20, 2022, 98 (89.1%) of 110 patients had at least 1 TRAE. **Table 5** presents the TRAEs associated with fruquintinib coupled with PD-1 inhibitors. A total of 13 (11.9%) patients suffered TRAEs of grade 3, and two patients suffered TRAE of grade 4. Two patients experienced two types of TRAEs of grade 3 or higher (one patient continued the fruquintinib dose reduction to 4 mg [hand-foot syndrome and hypertension], the other improved after symptomatic treatment and continued to receive the combination therapy [leukopenia and neutropenia]). The following were the most common TRAEs: thrombocytopenia (32.7%), elevated aspartate aminotransferase (AST) levels (28.2%), elevated alanine aminotransferase (ALT) levels (24.5%), diarrhea (23.7%), elevated thyroid-stimulating hormone (TSH) levels (22.7%), proteinuria (21.8%), hypothyroidism (20.0%), hyperlipidemia (19.1%), anemia (18.2%), leukopenia (16.4%), hypertension (15.5%), hand-foot syndrome (14.5%), elevated lactate dehydrogenase levels (14.5%), constipation (12.7%), nausea or vomiting (11.8%), and elevated bilirubin levels (10.9%). The most frequent grade 3 TRAE was hypertension. No deaths occurred as a result of the treatment.

**Table 5:** Treatment-related adverse events.

Adverse events	Any grade	Grade 1	Grade 2	Grade 3	Grade 4
Hand-foot syndrome	16 (14.5%)	9 (8.2%)	5 (4.5%)	2 (1.8%)	0 (0.0%)
Rash	3 (2.7%)	0 (0.0%)	3 (2.7%)	0 (0.0%)	0 (0.0%)
Hoarseness	3 (2.7%)	0 (0.0%)	3 (2.7%)	0 (0.0%)	0 (0.0%)
Nausea/vomiting	13 (11.8%)	13 (11.8%)	0 (0.0%)	0 (0.0%)	0 (0.0%)
Diarrhea	26 (23.7%)	8 (7.3%)	18 (16.4%)	0 (0.0%)	0 (0.0%)
Constipation	14 (12.7%)	10 (9.1%)	4 (3.6%)	0 (0.0%)	0 (0.0%)
Hypertension	17 (15.5%)	4 (3.6%)	10 (9.0%)	3 (2.7%)	0 (0.0%)
Leukopenia	18 (16.3%)	11 (10.0%)	5 (4.5%)	2 (1.8%)	0 (0.0%)
Neutropenia	6 (5.4%)	2 (1.8%)	2 (1.8%)	2 (1.8%)	0 (0.0%)
Thrombocytopenia	36 (32.7%)	24 (21.8)	10 (9.1%)	1 (0.9%)	1 (0.9%)
Anemia	20 (18.1%)	15 (13.6%)	4 (3.6%)	1 (0.9%)	0 (0.0%)
Proteinuria	24 (21.8%)	9 (8.2%)	14 (12.7%)	1 (0.9%)	0 (0.0%)
alanine aminotransferase elevated	27 (24.5%)	23 (20.9%)	4 (3.6%)	0 (0.0%)	0 (0.0%)
aminotransferase elevated	31 (28.1%)	27 (24.5%)	2 (1.8%)	2 (1.8%)	0 (0.0%)
Bilirubin elevated	12 (10.9%)	10 (9.1%)	1 (0.9%)	1 (0.9%)	0 (0.0%)
Hyperlipidemia	21 (19.1%)	21 (19.1%)	0 (0.0%)	0 (0.0%)	0 (0.0%)
Fatigue	7 (6.4%)	7 (6.4%)	0 (0.0%)	0 (0.0%)	0 (0.0%)
Oral mucositis	6 (5.4%)	2 (1.8%)	4 (3.6%)	0 (0.0%)	0 (0.0%)
Creatinine elevated	6 (5.4%)	6 (5.4%)	0 (0.0%)	0 (0.0%)	0 (0.0%)
Arrhythmia	1 (0.9%)	0 (0.0%)	1 (0.9%)	0 (0.0%)	0 (0.0%)
Lactate dehydrogenase elevated	16 (14.5%)	16 (14.5%)	0 (0.0%)	0 (0.0%)	0 (0.0%)
Hypothyroidism	22 (20.0%)	16 (14.5%)	6 (5.5%)	0 (0.0%)	0 (0.0%)
thyroid-stimulating hormone elevated	25 (22.7%)	20 (18.2%)	5 (4.5%)	0 (0.0%)	0 (0.0%)
Hyperthyroidism	2 (1.8%)	1 (0.9%)	1 (0.9%)	0 (0.0%)	0 (0.0%)
Adrenal cortex hypofunction	1 (0.9%)	0 (0.0%)	1 (0.9%)	0 (0.0%)	0 (0.0%)
Immune-associated pneumonia	1 (0.9%)	0 (0.0%)	1 (0.9%)	0 (0.0%)	0 (0.0%)
Joint pain	3 (2.7%)	3 (2.7%)	0 (0.0%)	0 (0.0%)	0 (0.0%)
Bleeding	6 (5.4%)	6 (5.4%)	0 (0.0%)	0 (0.0%)	0 (0.0%)
Hyperuricemia	10 (9.1%)	10 (9.1%)	0 (0.0%)	0 (0.0%)	0 (0.0%)

Eleven patients discontinued treatment due to TRAEs: proteinuria (2), rash (2), hypertension, hand-foot syndrome, oral mucositis, thrombocytopenia, diarrhea, arrhythmia, adrenal cortex hypofunction, and immune-associated pneumonia. Three patients experienced fruquintinib dose reduction: two with the hand-foot syndrome and one with hypertension.

As of January 20, 2022, 33 patients were still on treatment, and three were assessed to continue benefiting from the combination therapy and continued treatment after disease progression. Six patients discontinued treatment due to TRAEs, and 71 patients discontinued treatment due to disease progression.

## Discussion

MSI-H/dMMR CRC stimulates tumor antigen synthesis by increasing TMB, resulting in increased T-cell infiltration in the TME and responding well to ICIs (11, 12). In contrast, MSS/pMMR CRC with a TMB-L and limited T-cell infiltration were resistant to ICIs. The ORR of pembrolizumab in MSI-H mCRC was 40% in the KEYNOTE-016 study; however, it was 0% for MSS mCRC (4). Only 1 of 20 MSS/pMMR CRC patients treated with nivolumab plus ipilimumab showed an objective response in CHECKMATE 142 (13). New treatments to break through immunological resistance in MSS/pMMR CRC are urgently needed.

Tumor angiogenesis promotes tumor growth and metastasis while also constructing an immunosuppressive TME resistant to immunotherapy (7). By inhibiting VEGFR, fruquintinib normalizes tumor blood vessels, improves the TME’s hypoxic environment, and stimulates T-cell infiltration, all of which help reverse the immunosuppressive microenvironment. Fruquintinib shown significant selectivity for tumor cells and good antitumor activity in both preclinical and clinical studies (5, 14). Many studies have confirmed the mutual synergy of anti-angiogenesis inhibitors and ICIs in recent years. In a case report, fruquintinib combined with sintilimab in a patient with MSS/pMMR CRC who progressed on multiple lines of therapy brought about rapid remission. In mouse models, fruquintinib combined with a PD-1 inhibitor efficiently suppressed tumor growth in a syngeneic MSS-CRC model, enhancing antitumor immune response by decreasing the number of Treg cells and increasing tumor cell immunogenicity and T-cell infiltration (8). The ORR of regorafenib with nivolumab in MSS/pMMR CRC patients was 33.3%, and the PFS was 7.9 months in the REGONIVO study (15). Basic experiments and clinical trials have given us confidence in exploring combination therapy strategies for MSS/pMMR CRC; however, real-world evidence on combination therapy is still rare.

Our study included 110 MSS/pMMR CRC patients who received the combination regimen of fruquintinib and PD-1 inhibitors. At the deadline (January 20, 2022), 13 patients had objective responses, and 64 were rated as SD. The ORR was 11.8% (95% CI: 6.4–18.2), the DCR was 70.0% (95% CI: 60.9–78.2), and the PFS of all patients was 5.4 months (95% CI: 4.0-6.8). Because of the short follow-up time, we could not judge the OS, which was one of our study’s shortcomings.

Clinical trials of the MSS/pMMR CRC combination therapy have been conducted to explore therapeutic strategies for breaking immune tolerance based on the surprising outcomes of the REGONIVO study (15). The ORR of regorafenib combined with toripalimab was 15.2% (5/33) in MSS/pMMR CRC patients in the REGOTORI study (16). The ORR was 25% (4/16) in another retrospective study of fruquintinib/regorafenib combined with camrelizumab (17). No promising combination therapy is without its challenges. The ORR of regorafenib combined with avelumab was 0% (0/40) in the REGOMUNE study (18). In another retrospective study, regorafenib combined with PD-1 inhibitors had no objective response in 23 MSS/pMMR CRC patients (19). The above indicates that selecting the right combination therapy is crucial for patients to benefit from it.

Although our ORR and PFS were lower than those in the REGONIVO study, we believe that attaining such results in the real world is gratifying and represents a significant breakthrough in the treatment of MSS/pMMR CRC. We suspect this is related to discrepancies between clinical trials’ rigorous inclusion criteria and the actual clinical population included. It is worth noting that in our study, a patient with liver and lung metastases and a *KRAS* mutation had continuous PR for 8.5 months before the medication was discontinued owing to a suspected malignant arrhythmia. The patient’s status was stable at the cutoff date, and the follow-up period was 22.4 months. Comparing the efficacy of ICI monotherapy and fruquintinib monotherapy in MSS/pMMR CRC (4, 5), the combined therapy significantly improved treatment efficacy; so, we are eagerly awaiting the benefits of fruquintinib coupled with PD-1 inhibitors for more cancer patients.

Previous research has revealed that patients without liver metastases have a higher incidence of CD8+ T-cell infiltration than those with them, implying that patients with liver metastases benefit less from ICIs due to a weakened antitumor immune response (20). In our study, we found that patients with liver metastases had significantly shorter PFS durations than those without liver metastases, which also confirms the abovementioned finding. Notably, patients with lung metastases had greater ORR than those with liver metastases and those without lung metastases. This is consistent with the findings of the REGONIVO and REGOTORI studies (15, 16). Before patients receive combination therapy, we think the presence or absence of liver metastases can be used as a predictor of treatment efficacy.

TMB is a biomarker for immunotherapy in multiple solid tumors. TMB-H is a biomarker for pembrolizumab-treated solid tumors that may inadvertently reflect the tumors’ capacity to produce neoantigens (21, 22). Our study showed that TMB-H patients had a longer PFS, but this difference was not statistically significant. Perhaps TMB≥10mut/Mb is not a reliable predictor of immunotherapy efficacy for all solid tumors. There is still debate on the TMB-H threshold for MSS/pMMR CRC. Critical issues with reliably detecting TMB in a particular cancer type and then determining an ideal TMB threshold (if one exists) must be resolved in order to better adopt TMB-H as a reliable clinical biomarker. It also has to be determined whether TMB-H can be utilized as a biomarker for immunotherapy in conjunction with targeted therapy.

In clinical practice, ICIs are routinely administered with other drugs. However, few clinical trials have revealed predictors of the success of combination therapy. We won’t be able to choose patients who will benefit from combination therapy and maximize patient effectiveness unless we can identify the appropriate predictors. For this, we did some exploration. In 1878, Billroth discovered that tumor cells were wrapped in the thrombus formed by FIB through the body’s coagulation system to escape the killing effect of the body’s immune system (23). High levels of plasma FIB and D-Dimer have been associated with cancer metastasis, recurrence, and poor prognosis in a number of studies (24). The strongest associations of D-Dimer and FIB with poor prognosis in CRC were observed in a meta-analysis (25). In our study, it was determined that the level of FIB was associated with the PFS of combination therapy. However, the effect of D-Dimer level on efficacy is only meaningful in univariate analysis, we think it may be due to insufficient sample size. ALPase is highly expressed in tumor cells and can stimulate tumor cell growth, the elevated ALP levels in preoperative CRC patients are related to a poor prognosis (26). ALP levels were observed to correlate with PFS in MSS/pMMR CRC receiving combination therapy in our study. It is possible that liver injury caused by liver metastases caused us to observe such differences, but patients with elevated ALP levels in the without liver metastases group had poorer prognosis. ALP levels have been shown to be associated with radiologic undetectable occult metastasis in liver or bone tissue (27), and we think it still has value in judging the prognosis of MSS/pMMR CRC receiving combination therapy. Unfortunately, we are unable to study the underlying mechanism. In clinical practice, the detection of ALP and FIB is convenient, fast and economical, and we believe that it is feasible as a predictor of the efficacy of combined therapy.

In addition to the DNA mismatch-repair system, the other most important pathway involved in CRC development is the epidermal growth factor receptor (EGFR) signaling pathway including *KRAS* and *BRAF* mutations (28). *RAS* mutations are associated with a poor prognosis with EGFR inhibitor therapy (29). There was no significant difference in the PFS between *RAS* mutant patients and *RAS* wild patients in our study (4.1 months vs. 5.1 months, P>0.05). It remains to be seen if anti-EGFR therapy combined with ICIs can conquer *RAS* resistance to anti-EGFR therapy. According to previous studies, patients with *BRAF* mutations who progress after first-line therapy are less likely to receive later-line therapy and have a worse prognosis (30). Although only six patients had *BRAF* mutations in our study, two patients achieved PR, and the remaining four achieved disease control. The ORR was 33.3%, the DCR was 83.3%, and the PFS was 13.3 months. A high response rate of 50% was also observed in two patients with *BRAF* mutations in the REGOTORI study (16). According to previous reports, this may be related to PD-1 inhibitors altering *BRAF* abundance and increasing *BRAF* mutation-associated tumor-associated lymphocytes *via* the mitogen-activated protein kinase (MAPK) pathway (31, 32). Our study, despite its small sample size, proposes a novel therapeutic strategy for *BRAF*-mutant patients in the late stage. More large-scale randomized controlled studies are needed to substantiate the efficacy of fruquintinib coupled with PD-1 inhibitors in *BRAF*-mutant patients.

Safety was evaluated as a secondary endpoint in our study. Only 11 of the 110 patients discontinued treatment due to TRAEs, and the majority of them were able to continue treatment once the TRAEs were attenuated. The dose of fruquintinib was modified in three patients due to TRAEs, and all of them continued treatment after the dose of the drug was decreased. Although 89% of patients experienced TRAEs, the majority of these TRAEs were of grade 2 or lower (87%). It is worth noting that 10 patients developed hyperuricemia and six developed bleeding in our study; however, all of them recovered after symptomatic treatment. Due to suspected malignant arrhythmia, immune-related pneumonia, and immune-related adrenal insufficiency, three patients discontinued treatment. Immune-related adrenal insufficiency resolved with glucocorticoid therapy. The most common TRAEs are consistent with those seen with single-drug fruquintinib and single-drug PD-1 inhibitors (33, 34). Compared with the REGONIVO study, the overall incidence of TRAEs (100% vs. 89.1%) and the incidence of grade 3 TRAEs (20% vs. 11.8%) were lower (15). The following are some plausible explanations: 1. Doctors will adjust the dosage and usage of the drug in time according to the patient’s tolerance in the process of clinical management, which minimizes the risk of TRAEs to a certain extent; 2. In retrospective studies, the limitations of restricted data collection and incomplete data may decrease the incidence of TRAEs. 3. The dose expansion of regorafenib may have increased the incidence of TRAEs in the REGONIVO study.

This study had some limitations. First, being a retrospective study, it was prone to biases due to limited data collection and incomplete data, resulting in biased results. Second, the OS could not be determined because of the short follow-up duration and inadequate data collected. Third, infiltration of cytotoxic T-cells in tumors has been shown to predict immunotherapy response in different studies. Most patients have not been checked for PD-1/PD-L1 expression levels or T-lymphocyte values to reduce the burden of clinical treatment; as such, we could not investigate whether they can be used as biomarkers associated with combination therapy. Fourth, although we preliminarily identified liver metastases, ALP level, and FIB level as efficacy predictors for fruquintinib coupled with PD-1 inhibitors in MSS/pMMR CRC, the underlying mechanisms were not investigated. Fifth, our study used a variety of PD-1 inhibitors and was unable to determine the best medication combination. However, this may imply that we can select a more appropriate medicine combination for the patient based on their financial situation and tolerance without worrying about the drug’s effectiveness. Further large-scale randomized controlled trials are needed to determine the best combination therapy regimen.

In conclusion, MSS/pMMR CRC resistant to immunotherapy can benefit from combination therapy, and liver metastases, ALP levels, and FIB levels may indicate prognosis.

## Data availability statement

The original contributions presented in the study are included in the article/supplementary material. Further inquiries can be directed to the corresponding author.

## Ethics statement

The studies involving human participants were reviewed and approved by The First Affiliated Hospital of Zhengzhou University, Research and Clinical Experiment Ethics Office. Written informed consent for participation was not required for this study in accordance with the national legislation and the institutional requirements.

## Author contributions

WZ conceived the idea, designed, and supervised the study. ZZ, SL, DL, ZM, and LX conducted the study. WZ, ZZ, and SL analyzed the data. WZ and ZZ wrote the manuscript. All authors have read and agreed on the published version of the manuscript.

## Funding

This study was funded by Henan Province Medical Science and Technology Research Program (Provincial and Ministry Co-construction) Project (grant number SBGJ202003026) and Beijing Xisike Clinical Oncology Research Foundation.

## Acknowledgments

We thank Prof. Shuiling Jin for the insightful suggestions. We would like to thank Charlesworth (https://www.cwauthors.com.cn/) for English language editing. We also sincerely thank the editors and reviewers for their careful review and valuable opinions that helped to greatly improve the manuscript.

## Conflict of interest

The authors declare that this study was conducted in the absence of any commercial or financial relationships that could be construed as a potential conflict of interest.

## Publisher’s note

All claims expressed in this article are solely those of the authors and do not necessarily represent those of their affiliated organizations, or those of the publisher, the editors and the reviewers. Any product that may be evaluated in this article, or claim that may be made by its manufacturer, is not guaranteed or endorsed by the publisher.
